# Targeted myocardial gene expression in failing hearts by RNA sequencing

**DOI:** 10.1186/s12967-016-1083-6

**Published:** 2016-11-25

**Authors:** Kajari Dhar, Alexandra M. Moulton, Eric Rome, Fang Qiu, Jeff Kittrell, Eugenia Raichlin, Ronald Zolty, John Y. Um, Michael J. Moulton, Hesham Basma, Daniel R. Anderson, James D. Eudy, Brian D. Lowes

**Affiliations:** 1 Department of Internal Medicine, University of Nebraska Medical Center, 982265 Nebraska Medical Center, Omaha, NE 68198-2265 USA; 2 Department of Cardiothoracic Surgery, University of Nebraska Medical Center, Omaha, USA; 3 Department of Biostatistics, University of Nebraska Medical Center, Omaha, USA; 4 Department of Genetics, Cell Biology and Anatomy, University of Nebraska Medical Center, Omaha, USA

## Abstract

**Background:**

Myocardial recovery with left ventricular assist device (LVAD) therapy is highly variable and difficult to predict. Next generation ribonucleic acid (RNA) sequencing is an innovative, rapid, and quantitative approach to gene expression profiling in small amounts of tissue. Our primary goal was to identify baseline transcriptional profiles in non-ischemic cardiomyopathies that predict myocardial recovery in response to LVAD therapy. We also sought to verify transcriptional differences between failing and non-failing human hearts.

**Methods:**

RNA was isolated from failing (n = 16) and non-failing (n = 8) human hearts. RNA from each patient was reverse transcribed and quantitatively sequenced on the personal genome machine (PGM) sequencer (Ion torrent) for 95 heart failure candidate genes. Coverage analysis as well as mapping the reads and alignment was done using the Ion Torrent Browser Suite™. Differential expression analyses were conducted by empirical analysis of digital gene expression data in R (edgeR) to identify differential expressed genes between failing and non-failing groups, and between responder and non-responder groups respectively. Targeted cardiac gene messenger RNA (mRNA) expression was analyzed in proportion to the total number of reads. Gene expression profiles from the PGM sequencer were validated by performing RNA sequencing (RNAseq) with the Illumina Hiseq2500 sequencing system.

**Results:**

The failing sample population was 75% male with an average age of 50 and a left ventricular ejection fraction (LVEF) of 16%. Myosin light chain kinase (MYLK) and interleukin (IL)-6 genes expression were significantly higher in LVAD responders compared to non-responders. Thirty-six cardiac genes were expressed differentially between failing and non-failing hearts (23 decreased, 13 elevated). MYLK, Beta-1 adrenergic receptor (ADRB1) and myosin heavy chain (MYH)-6 expression were among those significantly decreased in failing hearts compared to non-failing hearts. Natriuretic peptide B (NPPB) and IL-6 were significantly elevated. Targeted gene expression profiles obtained from the Ion torrent PGM sequencer were consistent with those obtained from Illumina HiSeq2500 sequencing system.

**Conclusions:**

Heart failure is associated with a network of transcriptional changes involving contractile proteins, metabolism, adrenergic receptors, protein phosphorylation, and signaling factors. Myocardial MYLK and IL-6 expression are positively correlated with ejection fraction (EF) response to LVAD placement. Targeted RNA sequencing of myocardial gene expression can be utilized to predict responders to LVAD ther*a*py and to better characterize transcriptional changes in human heart failure.

## Background

Left ventricular assist devices are commonly being utilized to treat patients with advanced heart failure [1]. Mortality due to infection, bleeding, stroke, right heart failure and device malfunction continues as a significant problem in this population but increasingly patients are receiving these devices as destination therapy [1]. Cardiac transplantation is not an option for the majority of these patients due to limitations in donor supply [2]. Myocardial recovery with LVAD therapy to the degree that allows device explant remains relatively rare for these patients [1, 3]. LVAD therapy may be associated with myocardial atrophy, changes in transcriptional profiles, and persistent abnormalities in protein phosphorylation [4, 5].

There is however increasing evidence that a subset of patients with LVAD therapy may remodel and recover sufficient myocyte function to allow device explant. Utilizing ventricular unloading, myocardial conditioning and guideline directed heart failure medical therapy it may feasible to explant a significant number of patients with LVADs [6]. Myocardial recovery, while unusual in ischemic cardiomyopathies, may occur in 20–25% of non-ischemic cardiomyopathy patients [7].

Heart failure is characterized on a molecular level by transcriptional changes involving adrenergic signaling mechanisms, calcium handling, contractile proteins, inflammation, and metabolism [8]. Next-generation sequencing allows for gene expression quantification, the study of epigenetic effects, identification of signal nucleotide polymorphisms and measurement of transcriptional variations. Understanding the transcriptome is essential to understanding disease development, progression, and response to therapeutics. Transcriptional profiling is currently being used to guide treatment in many forms of cancer and to diagnose cardiac rejection. It is unknown if gene expression profiles can predict response to LVAD therapy. Our primary goal was to identify transcriptional profiles in non-ischemic cardiomyopathy patients that predict myocardial recovery. We also sought to verify transcriptional differences between failing and non-failing human hearts utilizing targeted non-optical sequencing.

## Methods

This study was approved by the IRB at the University of Nebraska Medical Center. Failing tissue was obtained from non-ischemic cardiomyopathy patients (n = 16) who met standard criteria for LVAD placement either as destination therapy or as bridge to transplant. Tissue was obtained at the time of LVAD placement and stored in RNA later prior to freezing at −80 °C. Non-failing tissue was obtained by endomyocardial biopsy in transplant patients with normal cardiac function during routine surveillance (n = 8). Cardiac function was evaluated by echocardiography.

### Isolation of RNA

RNA was isolated from approximately 10–20 mg of cardiac tissue using the RNeasy^®^ Mini Kit (Qiagen^®^). Quantitative measurement of the RNA was taken using Qubit^®^ RNA assay kit by Qubit 2.0 fluorometer (Invitrogen, Life Technologies).

### Constructing RNA Library

Ion AmpliSeq™ RNA Library Kit and Custom Panels were utilized in the construction of RNA library for this investigation, and all experiments were carried out in accordance with the instructions from the manufacturer (Ion Torrent, Life Technologies). 10 ng of RNA, isolated from the cardiac tissue, is reverse transcribed to synthesize complementary deoxyribonucleic acid (cDNA) using the Ion AmpliSeq™ RNA RT Module and Applied Biosystems thermal cycler. Next target sequences were amplified using Ion AmpliSeq™ RNA Custom Panels and Library Kit (life technologies). Our custom panel was designed to target 95 cardiac genes we previously have associated with reverse remodeling in heart failure utilizing the Ion AmpliSeq™ Designer [8]. After amplification of target sequences, primer sequences were partially digested using FuPa reagent from Ion AmpliSeq™ RNA Library Kit. Ion AmpliSeq™ adaptors were then ligated to the targeted deoxyribonucleic acid (DNA) and purification of those DNA fragments followed. Target DNA sequences were purified in a two-round purification process with Dynabeads^®^ Magnetic Beads where surplus primer sequences and high-molecular weight DNA are isolated and discarded from the solution. Following purification of the library, the library is amplified using Ion AmpliSeq™ RNA Library Kit and further purified using single-round purification with Dynabeads^®^ Magnetic Beads. After amplification and purification of the library, Qubit^®^ 2.0 fluorometer was used with Qubit^®^ dsDNA HS Assay Kit to quantitatively measure the DNA library.

### Preparation of targeted DNA template

Amplified stock library was diluted for appropriate Ion library preparation. Ion One Touch™ 2 System was utilized to amplify individual diluted libraries via emulsion polymerase chain reaction (PCR) on ion sphere particles (ISPs). The template-positive ISPs were then enriched using Ion One Touch™ Enrichment System.

### Sequencing

Sequencing was performed for all patients on the Ion Torrent PGM utilizing the Ion 316 chip. Coverage analysis as well as mapping the reads and alignment was done using the Ion Torrent Browser Suite™. Gene expression profiles from the PGM sequencer were validated with a subsequent population by performing RNAseq with the Illumina Hiseq2500 sequencing system. RNAs from 7 non failing and 3 failing samples were sequenced using the Illumina HiSeq2500 sequencing system (University of Nebraska Medical Center, DNA core facility). All paired end sequencing adaptors were trimmed using fqtrim and any reads shorter than 36 bases were discarded. A quality filter using fqtrim −q 5 was then applied to the reads. Paired end reads that passing those filters were mapped to Homo sapiens (release 37) reference sequence (GRCh37/hg19); using BowTie2 and TopHat2. The raw read counts per gene were generated using the Rsubread package.

### Statistical analysis

For the LVAD responder analysis the failing heart patients were divided into two groups: responders (∆LVEF ≥ 20%) and non-responders (∆LVEF < 20%). The expression of each gene on the cardiac panel was assessed in proportion to the total reads on the chip. The differential expression analysis was conducted using edgeR package in bioconductor developed by Robinson et al. [9, 10]. Differential gene expression between the non-failing heart patients (n = 8) and failing heart patients (n = 16) was analyzed in a similar fashion. The gene Calreticulin 3 (CALR3) was removed from the data due to 21 samples had zero counts for this gene. Spearman’s correlation coefficients were calculated to evaluate inter-platform consistency for raw counts and normalized counts per million (cpm) across two platforms (Ion Torrent and Illumina). The normalization factors for the data were estimated by the trimmed mean of the M-values normalization method in the edgeR package and used to adjust for varying sequencing depths and potentially other technical effects across samples.

To further evaluate the biological pathways, log fold change utilizing Ingenuity Pathway Analysis (IPA) was carried out on the targeted panel genes between failing and non-failing samples.

## Results

### Patient characteristics

**Fig. 1:**
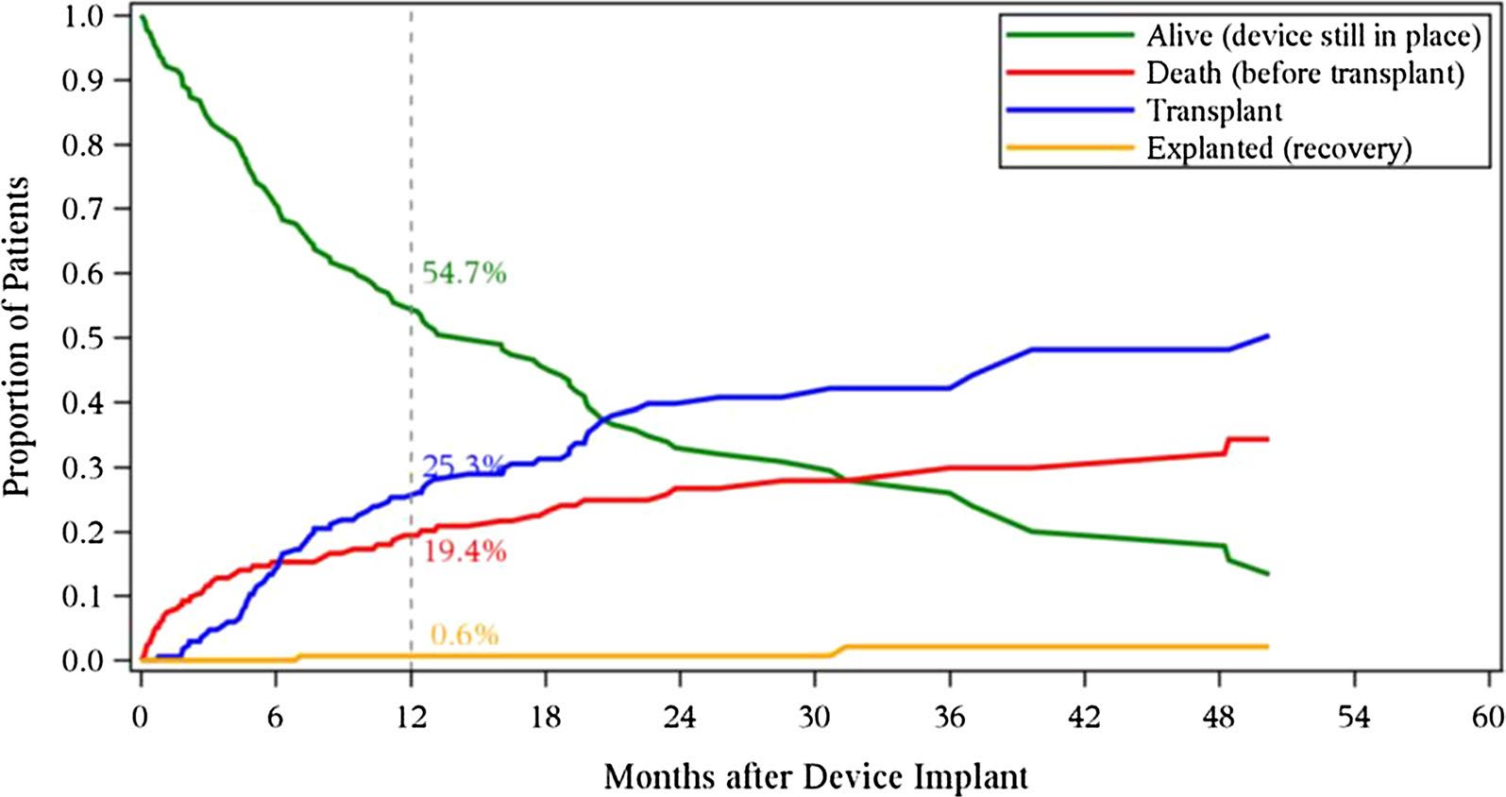
Competing outcomes for continuous flow LVADs (University of Nebraska, June 23 2006 to June 30 2016, n = 177)

We analyzed a total 34 of human heart samples, 16 failing, and 8 non-failing. Patient clinical characteristics are shown on Table 1. The failing sample population was 75% male with an average age 50. The failing patients had a New York Heart Association (NYHA) functional classification of III-IV and diabetes mellitus was common. EFs were measured before and after 3 months LVAD implantation. Change in ejection fraction (ΔEF) greater than 20 signifies responder after LVAD placement. By these criteria, 10 patients were non responders and 6 patients were responders. The non- failing patients had EFs ranging from 60 to 65% whereas the EFs of LVAD patients were 15–25% at the time of implant. All patients in this study were recipients of the Heartmate II LVAD at the University of Nebraska Medical Center. Our center implanted 177 LVADs between June of 2006 and June of 2016. Most of these implants (55%) were for critical cardiogenic shock or a progressive declining clinical status despite inotropic support. The remainder were stable on inotropic therapy or with advanced resting symptoms. The overall clinical outcomes of LVAD therapy are shown in Fig. 1. Overall survival with LVAD as bridge to transplant or destination therapy at one year is approximately 80%. Very few patients historically have been explanted for recovery.

**Table 1:** Patient characteristics

Variable	Non-failing hearts, n = 8	Failing hearts, n = 16
Age (years)	50 ± 13.30	50 ± 16.43
Male (%)	75%	75%
Caucasian/AA/hispanic-Asian (%)	12.5/75/12.5	70/20/10
Comorbid illness (%)	DM 62.5%	DM50%/HTN87.5%/Afb44%?CKD20%
LVEF at presentation (%)	60 ± 4.63	15.75 + 10.75
NYHA class	N/A	3.68 + 0.60
Outcome (%)	N/A	LVAD in place 50%/heart transplant 44%/deceased 6%

### Responder vs non-responder RNA-Seq

**Fig. 2:**
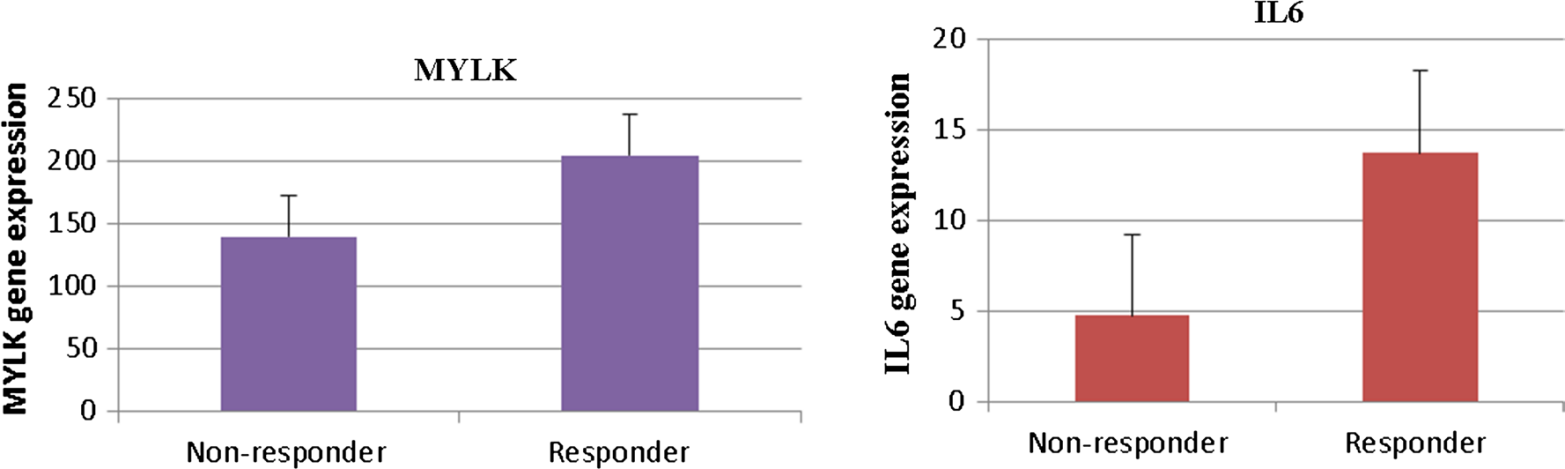
Gene expression levels associated with myocardial recovery. (ΔEF > 20 signifies responder to LVAD therapy. MYLK and IL6 expression was highly significant with p ≤ 0.005 and p ≤ 0.0002 respectively)

Our study showed that MYLK (p = 0.005) and IL 6 (p < 0.0002) expression were significantly higher in LVAD responder patients than non-responder patients, Fig. 2. MYLK gene or myosin light chain kinase activates and regulates myosin light chain in the heart by phosphorylation. MYLK is necessary for normal cardiac function. Down regulation of MYLK expression increases the risk of heart failure [11–14]. Levels of IL-6 are found considerably higher in myocardium and serum of patients receiving LVAD and with advanced heart failure [15–17]. Further analysis of an additional 5 patients pre and post LVAD who did not recover cardiac function and required transplant shows that LVAD therapy decreased myocardial IL-6 (log fold change −2, p = 0.004) but did not change MYLK (p = 0.6).

### Failing vs non-failing RNA-Seq results

**Fig. 3:**
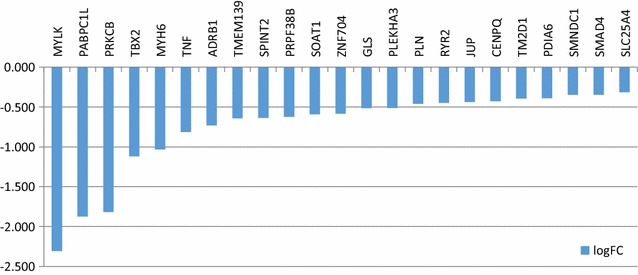
Down regulated genes in failing myocardium (p < 0.05, log2-fold-change of expression for failing vs. non-failing)

**Fig. 4:**
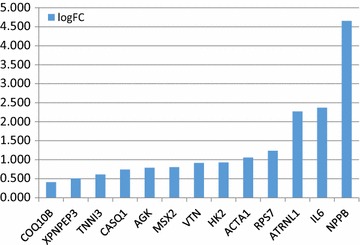
Up regulated genes in failing myocardium (p < 0.05, log2-fold-change of expression for failing vs. non-failing)

Here we tried to understand the changes of cardiac genes expression during heart failure in association with contractile dysfunction. We evaluated mRNA expression of 95 cardiac gene using RNA-Sequence technology. It was found that 36 genes were differentially expressed when we compared failing versus non failing hearts (p < 0.05). Twenty-three genes were significantly decreased in failing hearts including MYLK, ADRB1 and MYH6, Fig. 3. In contrast 13 genes were up-regulated including NPPB and IL-6, Fig. 4. A complete list of genes studied, p values and false discovery rates [18] are listed in Appendix.

### Functional and pathway analysis in IPA

**Fig. 5:**
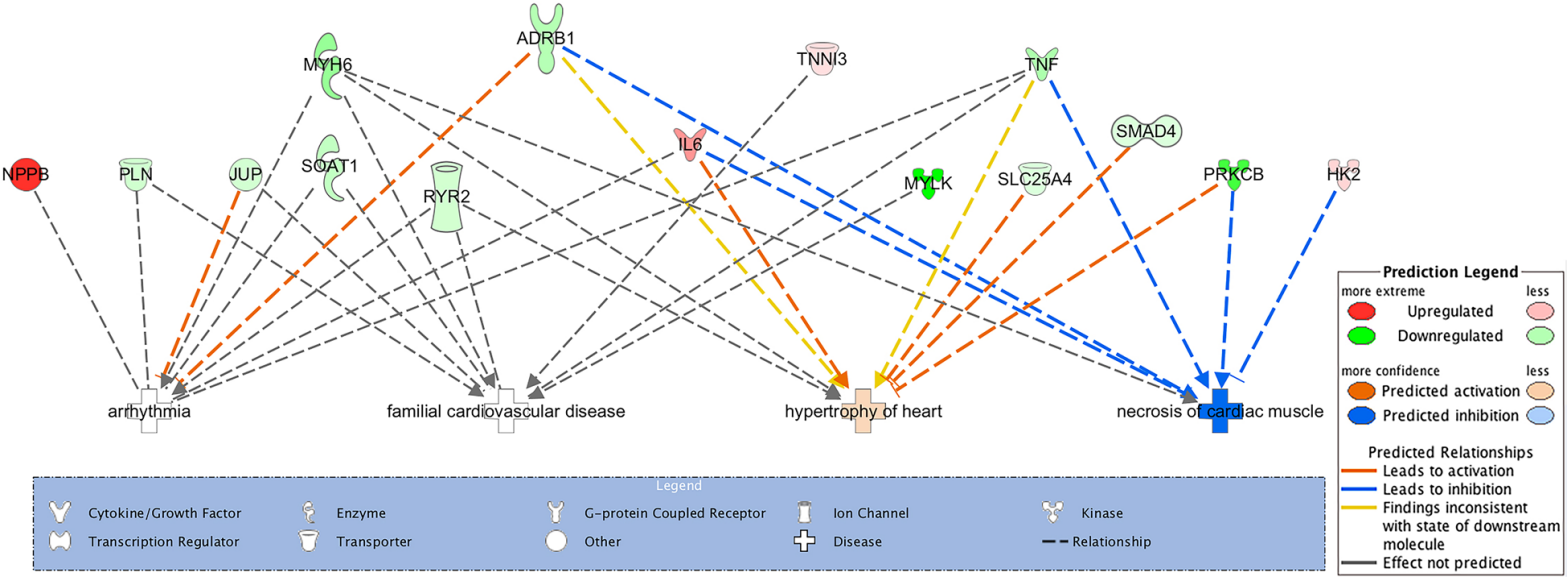
Ingenuity Pathway Analysis of log-fold change of genes in failing compared to non-failing

To further analyze the biological pathways, log fold change utilizing IPA was carried out on the targeted panel genes between failing and non-failing hearts. The differentially expressed genes involved in cardiomyopathy and muscle formation were plotted. The top canonical pathways, molecular functions, upstream regulators and toxic effects are shown in Table 2. Figure 5 illustrates the network for genes that were differentially expressed, their molecular function and their relationship to different diseases including arrhythmia, hypertrophy of the heart, familial heart disease and necrosis of cardiac muscles. As shown in Fig. 5, eight genes of the targeted panel were involved in hypertrophy of the heart. Four genes clearly lead to activation of this pathway, up-regulation of IL-6 (an activator) and down regulation of SMAD4, PRKCB and SLC25A4 (inhibitors). These molecular changes collectively augment the hypertrophy signal of the heart. Six genes of the targeted panel were involved in the necrosis of cardiac muscle with a net z score of −2.115. Up-regulation of HK2 and IL6 (known inhibitors) and down regulation of ADRB1, PRKCB and TNF (known activators). The net effect of these changes are predicted to inhibit necrosis of the heart. Finally, nine molecules with a net of “not predicted effect” were involved in the familial cardiovascular disease and arrhythmia that could be furthered studied.

**Table 2:** IPA analysis

Name	p value
Top canonical pathways	
Calcium signaling	9.78E−06
Nitric oxide signaling in the cardiovascular system	2.07E−05
Protein kinase A signaling	3.37E−05
Tight junction signaling	1.53E−04
Hepatic fibrosis/hepatic stellate cell activation	2.17E−04
Top upstream regulators	
IKBKG	3.25E−08
Phenylephrine	3.50E−08
Fucoidin	9.83E−08
GATA4	1.63E−07
SLC16A3	2.89E−07
Molecular and cellular functions	
Cell morphology	1.49E−03 to 1.70E−08
Cellular movement	1.40E−03 to 3.96E−08
Gene expression	1.09E−03 to 3.96E−08
Cell death and survival	1.57E−03 to 1.29E−07
Cell-to-cell signaling and interaction	1.49E−03 to 2.21E−07
Top tox lists	
Cardiac hypertrophy	2.16E−08
Cardiac necrosis/cell death	4.44E−06
Increases heart failure	6.87E−06
Cardiac fibrosis	1.45E−05
Hepatic fibrosis	2.16E−05

### RNA-Seq validation

To validate the results obtained from Ion torrent PGM sequencer by RNAseq analysis, Illumina HiSeq sequencing was utilized. Ten patients were sequenced for selected 95 genes using RNAseq method on both sequencing platforms (Illumina HiSeq and Ion Torrent). Illumina data were processed to obtain raw read counts for genes by BowTie, TopHat, and Rsubread packages respectively [19–21]. To evaluate inter-platform consistency for gene expression measures, we computed Spearman’s correlation between raw counts and normalized cpm from two platforms for each individual, and the average spearman’s correlation across 10 individuals. The results demonstrated high inter-platform consistency for gene expression measures across two platforms. (Spearman R ≥ 0.83 for each patient sample, and average Spearman R = 0.84 using raw counts. Spearman R ≥ 0.84 for each patient sample and average Spearman R = 0.86 using normalized cpm).

## Discussion

The main findings of this study are that MYLK2 is diminished in heart failure, predicts response to LVAD therapy, and does not improve in advanced heart failure patients post LVAD who require transplantation. Our results further characterize transcriptional changes between failing and non-failing human hearts and identify gene expression profiles associated with myocardial recovery on LVAD therapy. Heart failure is a heterogeneous disease process. Etiology, disease duration, cardiac remodeling, medical therapy, and molecular signatures have all been associated with myocardial recovery in non-ischemic cardiomyopathies. Recent studies suggest that up to 20 percent of patients may recover their cardiac function within their first year post LVAD [22]. Molecular phenotyping is increasingly being utilized as a clinical tool to risk stratify patients and guide clinical decision making. Transcriptional profiles are being utilized to screen heart transplant patient for rejection, identify patients with coronary disease, and guide treatment decisions for cancer patients. It may be feasible utilizing clinical characteristics and molecular markers to risk stratify patients for early transplant versus more prolonged aggressive attempts to recover myocardial function with medical and device therapies. Previous studies have identified myocardial microRNA (miRNA) signatures as potential biomarkers of recovery. Our results suggest that mRNA patterns may also be helpful in risk stratifying patients undergoing LVAD therapy.

Baseline MYLK and IL-6 levels were both abnormal in failing myocardium and associated with response to LVAD therapy. Myosin is comprised of two heavy chain and two pairs of light chains. Myosin light chain 2 (MLC-2) is a key regulatory component of myosin. Phosphorylation of MLC-2 is known to regulate both calcium sensitivity as well as sarcomere assembly [11, 23, 24]. MLC-2 can be phosphorylated by MYLK or protein kinase C and is dephosphorylated by light chain phosphatase. MLC-2 phosphorylation is known to be decreased in the end-stage failing human heart [25]. Our results would suggest that down-regulation of MYLK expression maybe contributing to this process. The association of higher levels of MYLK with improved myocardial recovery on LVAD therapy is consistent with prior evidence supporting this gene as an adaptive mechanism which facilities sarcomere organization in response to hypertrophic stimuli. Persistent abnormalities in MYLK post LVAD therapy in patients who did not recover are consistent with ongoing stress and injury in this population.

IL-6 is known to be elevated systemically in heart failure and levels have been associated with severity of disease [26]. Elevated expression of myocardial IL-6 in advanced heart failure could be contributing to this process. IL-6 can be produced by leukocytes, endothelial cells or vascular smooth muscle cells. Levels have been correlated with hemodynamic and neurohormonal variables. IL-6 is capable of producing myocardial dysfunction, vascular dilation, and muscle wasting. It is paradoxical that higher IL-6 levels at baseline are associated with enhanced recovery with LVAD therapy. IL-6 appears to be an important mediator in ventricular hypertrophy and does increase nitric oxide production [27]. These mechanisms may play an important role in adapting to heart failure. IL-6 is extremely inducible in response to IL-1, TNFα, viral infection and angiotensin II peptide. It acts through two distinct mechanisms. The first one is a classic membrane receptor initiated pathway and the second one is trans-signaling pathway. It has been shown that IL-6 induces and activates signal transducer and activator of transcription 3 (STAT3) gene by engaging the suppressor of cytokine signaling 3 (SOCS3) gene. Multiple studies showed that the activation of STAT3 promotes cardiomyocyte survival and hypertrophy, as well as cardiac angiogenesis [28]. Our results suggest LVAD therapy decreases myocardial IL-6 expression substantially even in patients who do not recover. Hence, it is unlikely to be contributing to the failure of myocardial recovery in patients post LVAD.

Heart failure is a complex molecular disease process involving more than IL-6 and MYLK. Numerous transcriptional changes have been described involving a complex gene network including contractile proteins, calcium handling, metabolism, signal transduction, apoptosis and inflammation [8, 29–31]. High throughput RNA sequencing now allows for the rapid quantification of gene expression in small amounts of tissue either globally or in a targeted fashion.

Most next generation sequencing technologies currently on the market use image capture, fluorescent detection or optical registration of some kind to capture sequencing data, which is then, converted to digital information. Our study utilized direct sequencing on a disposable semiconductor chip in a targeted fashion. This technology is relatively rapid, inexpensive and allows for quantification of mRNA over a broad range of gene expression levels. Between failing and non-failing human hearts we identify 36 genes that are differentially expressed. Other studies have previously identified these genes in association with heart failure and sequencing by optical and non-optical methodologies produced similar results. This suggests that RNA sequencing might provide a valuable tool to further characterize the molecular mechanisms of heart failure.

This study has several limitations. In regards to recovery, it is a single center retrospective analysis. There are several potential confounders that were not evaluated including genetic etiologies which could have impacted recovery independent of baseline gene expression. The study evaluated the transcriptome of only a limited number of genes. There are almost certainly other genes whose expression may be predictive of recovery or important mechanistically in heart failure. Finally, the transplant patients utilized as controls within this study may have different gene expression secondary to other factors such as immunosuppressive therapy independent of contractile dysfunction. For this reason we targeted candidate genes that previously have been identified in association with heart failure or myocardial remodeling.

While cardiac transplantation remains the gold standard for treating advanced heart failure, it is not an effective option for the majority of patients due to limitations in donor supply. The number of patients receiving left ventricular assist devices is growing exponentially. Less than 1% of patients are currently having their devices explanted for recovery. The rapid evolution in sequencing technologies may allow us to better classify LVAD patients who may recover. Prospective clinical trials to identify biomarkers of recovery are needed to help decision making in this population of patients. RNA signatures and clinical characteristics may ultimately help identify a subset of patients who can recover cardiac function.

## Conclusions

MYLK2 is diminished in heart failure, predicts response to LVAD therapy, and does not improve in advanced heart failure patients post LVAD who require transplantation. Targeted next generation RNA sequencing allows rapid quantitative characterization of the transcriptional changes that occur in heart failure. Changes in this gene network involves contractile proteins, calcium handling, metabolism, signal transduction, apoptosis and inflammation. Pathway analysis suggests that these changes ultimately contribute to cardiac hypertrophy, cardiac necrosis, cell death, increased heart failure risk and cardiac fibrosis.

## Appendix

See Table 3.

**Table 3:** Failing vs non-failing

Gene id	logFC	p value	FDR
PRKCB	−1.819	2.11E−11	1.98E−09
RPS7	1.239	6.93E−09	3.26E−07
ACTA1	1.059	3.94E−07	1.23E−05
PABPC1L	−1.873	1.22E−06	2.87E−05
ATRNL1	2.273	4.93E−06	9.27E−05
MYLK	−2.307	1.08E−05	0.00017
NPPB	4.653	1.47E−05	0.00020
IL6	2.371	6.33E−05	0.00074
ZNF704	−0.585	0.00015	0.0015
TBX2	−1.120	0.00049	0.0044
PRPF38B	−0.623	0.00051	0.0044
XPNPEP3	0.510	0.00176	0.014
SOAT1	−0.591	0.00210	0.015
VTN	0.919	0.00378	0.024
AGK	0.793	0.00425	0.024
HK2	0.928	0.00454	0.024
MSX2	0.806	0.00464	0.024
MYH6	−1.033	0.00471	0.024
ADRB1	−0.730	0.00486	0.024
TM2D1	−0.394	0.00570	0.027
PLEKHA3	−0.513	0.00611	0.027
CASQ1	0.743	0.00649	0.028
SMAD4	−0.347	0.00943	0.038
GLS	−0.515	0.00978	0.038
JUP	−0.437	0.010	0.038
SPINT2	−0.639	0.012	0.044
SMNDC1	−0.347	0.015	0.053
COQ10B	0.410	0.017	0.057
PLN	−0.461	0.018	0.059
TNNI3	0.613	0.021	0.067
PDIA6	−0.392	0.022	0.068
CENPQ	−0.429	0.024	0.070
TMEM139	−0.642	0.036	0.103
SLC25A4	−0.315	0.041	0.112
RYR2	−0.449	0.042	0.112
TNF	−0.814	0.048	0.125
C10orf88	−0.325	0.051	0.129
MYL2	0.544	0.055	0.135
MYOZ2	0.424	0.067	0.161
DSP	−0.369	0.068	0.161
SLC9B2	−0.391	0.075	0.170
ROBO4	−0.354	0.076	0.170
PPIC	0.351	0.081	0.177
DES	0.446	0.083	0.177
CETP	−0.727	0.101	0.212
TNNC1	0.355	0.117	0.234
NUFIP2	0.272	0.117	0.234
BAG3	0.308	0.120	0.235
TBL1XR1	0.227	0.136	0.261
STARD3	−0.275	0.152	0.286
ACTN2	0.289	0.172	0.318
DGAT1	−0.233	0.177	0.321
LAMP2	−0.240	0.183	0.323
SPCS3	−0.186	0.186	0.323
CAPZA2	−0.266	0.191	0.327
ERF	−0.233	0.209	0.351
UBE2B	−0.254	0.228	0.377
LDB3	−0.233	0.236	0.382
ACTC1	0.284	0.240	0.382
VCL	0.267	0.246	0.385
DSG2	−0.206	0.255	0.393
TAZ	−0.219	0.276	0.418
IL1B	−0.557	0.305	0.455
RPS18	0.198	0.311	0.457
ARHGEF15	−0.199	0.322	0.459
TMEM43	0.229	0.323	0.459
SGCD	−0.195	0.329	0.462
ATP6V1B2	0.145	0.343	0.474
PCDHB15	−0.220	0.361	0.492
MYBPC3	−0.156	0.377	0.506
RFXAP	−0.179	0.423	0.560
DSC2	−0.116	0.447	0.583
TPM1	0.171	0.464	0.597
WDR44	0.111	0.500	0.636
DMD	0.150	0.531	0.666
ME2	−0.100	0.564	0.697
RAP2B	−0.132	0.598	0.729
PKP2	0.126	0.612	0.737
ERCC4	0.078	0.648	0.771
PRKAG2	0.087	0.661	0.771
PHF2	−0.069	0.665	0.771
MAGEE1	−0.066	0.726	0.832
HIF1AN	−0.044	0.737	0.835
CAV3	0.052	0.761	0.851
ABCC9	−0.049	0.793	0.877
TNNT2	−0.044	0.821	0.898
MYH7	−0.021	0.920	0.983
LEMD3	−0.012	0.922	0.983
COX8A	0.018	0.935	0.983
IRF2	−0.008	0.949	0.983
MYL3	−0.007	0.958	0.983
FPGT	−0.002	0.977	0.983
BCL2L13	−0.002	0.980	0.983
PPM1D	0.008	0.983	0.983

## Abbreviations

LVAD: left ventricular assist device
RNA: ribonucleic acid
PGM: personal genome machine
edgeR: (empirical analysis of digital gene expression data in R)
mRNA: messenger RNA
RNAseq: RNA sequencing
LVEF: left ventricular ejection fraction
MYLK: myosin light chain kinase
IL: interleukin
ADRB1: Beta-1 adrenergic receptor
MYH-6: myosin heavy chain 6
NPPB: natriuretic peptide B
EF: ejection fraction
cDNA: complementary DNA
DNA: deoxyribonucleic acid
PCR: polymerase chain reaction
ISPs: ion sphere particles
ΔLVEF: change in left ventricular ejection fraction
CALR3: calreticulin 3
cpm: counts per million
IPA: Ingenuity Pathway Analysis
NYHA: New York Heart Association
ΔEF: change in ejection fraction
miRNA: microRNA
MLC-2: myosin light chain 2
STAT3: signal transducer and activator of transcription 3
SOCS3: suppressor of cytokine signaling 3
